# Protective Effect of Novel *Lactobacillus plantarum* KC3 Isolated from Fermented Kimchi on Gut and Respiratory Disorders

**DOI:** 10.3390/microorganisms11040967

**Published:** 2023-04-07

**Authors:** Min-Seon Park, Yu-Jeong Kim, Han-Jae Shin, Yoo Jin Kwon, Jaeryang Chu, Inock Lee, Kyung Hwan Kim, Byoung Kook Kim, Seung-Hyung Kim, Hwi Won Seo, Tae-Won Kim

**Affiliations:** 1 Infectious Disease Research Center, Korea Research Institute of Bioscience and Biotechnology, Daejeon 34141, Republic of Korea; 2 College of Veterinary Medicine (BK21 FOUR Program), Chungnam National University, Daejeon 34131, Republic of Korea; 3 Biosystems & Bioengineering Program, University of Science and Technology (UST), Daejeon 34113, Republic of Korea; 4 KT&G Research Institute, Daejeon 34128, Republic of Korea; 5 Chong Kun Dang Bio Research Institute (CKDBiO), Seoul 03722, Republic of Korea; 6 Institute of Traditional Medicine and Bioscience, Daejeon University, Daejeon 34520, Republic of Korea

**Keywords:** *Lactobacillus plantarum* KC3, probiotics, ambient particulate matter, lung inflammation, colitis

## Abstract

Probiotics have been shown to possess anti-inflammatory effects in the gut by directly reducing the production of pro-inflammatory cytokines and by secreting anti-inflammatory molecules. However, their systemic anti-inflammatory effects have not been thoroughly investigated. In this study, we aimed to develop probiotics that have efficacy in both intestinal and lung inflammation. *Lactobacillus plantarum* KC3 (KC3), which was isolated from kimchi, was selected as a pre-candidate based on its inhibitory effects on the production of pro-inflammatory cytokines in vitro. To further validate the effectiveness of KC3, we used ear edema, DSS-induced colitis, and ambient particulate-matter-induced lung inflammation models. First, KC3 exhibited direct anti-inflammatory effects on intestinal cells with the inhibition of IL-1β and TNF-α production. Additionally, KC3 treatment alleviated ear edema and DSS-induced colic inflammation, improving colon length and increasing the number of regulatory T cells. Beyond its local intestinal anti-inflammatory activity, KC3 inhibited pro-inflammatory cytokines in the bronchoalveolar fluid and prevented neutrophil infiltration in the lungs. These results suggest that KC3 could be a potential functional ingredient with respiratory protective effects against air-pollutant-derived inflammation, as well as for the treatment of local gut disorders.

## 1. Introduction

Lactic-acid-producing bacteria that were isolated from fermented kimchi have been shown to possess various health benefits, including immune enhancement and anti-cancer, anti-obesity, and anti-atherosclerosis properties [1,2]. Studies have primarily focused on immune-related potency by activating immune cells, preventing infection, and developing the immune system [3]. Recent research on the gut microbiome and probiotics has demonstrated that the gut microbiome plays a crucial role in interactions with almost all immune cells, including neutrophils, dendritic cells, macrophages, T cells, and B cells, suggesting that the gut microbiome–immune cell interaction involves immune modulation beyond just immune stimulation [4,5].

Recent studies have investigated the effects of probiotics on both local and systemic immune regulation through modulation of the gut microbiota. For example, probiotics could improve gut barrier function, reduce gut inflammation, and enhance the production of beneficial metabolites that can lead to improvements in local immune responses [6]. Additionally, probiotics have been shown to have effects on systemic immune function, such as enhancing natural killer cell activity, regulating cytokine production, and modulating T cell differentiation [7,8].

Although research on the relationship between probiotics and respiratory health is still in its early stages, there is growing evidence to suggest that probiotics could be relevant to the prevention and treatment of respiratory diseases. A previous clinical study found that administration of *L. plantarum* L-137 reduced airway infection and improved respiratory function [9]. Another study showed that the administration of a combination of *Lactobacillus* and *Bifidobacterium* probiotics reduced inflammation in the airways of patients with asthma [10]. Moreover, probiotics have also been found to enhance the effectiveness of conventional treatments for respiratory diseases. For instance, a study using a mouse model of asthma found that *L. rhamnosus* had a synergistic effect when combined with corticosteroid treatment in reducing airway inflammation [11]. A key factor of *Lactobacillus* probiotics in defending the respiratory tract against inflammation could be attributable to their immune regulatory effect. Colonized lactobacillus in the intestine could regulate the gut immune cell in mucosal lymphoid tissue, adjusting the balance between T-helper type 1 (Th1) cell-related immune response and Th2 response [12]. In addition, some probiotics could stimulate regulatory T (T_reg_) cells by modulation of mucosal dendritic cells enhancing the IL-10 and TGF-β secretion, which consequently shows anti-inflammatory potency and keeps the homeostatic environment, inhibiting the proinflammatory cytokines such as TNF-α and IFN-γ [13,14].

The potential of probiotics in alleviating respiratory inflammation led us to investigate the impact of probiotics on respiratory diseases caused by fine dust. Fine dust has been considered the main cause of a wide spectrum of respiratory damages, from acute respiratory distress syndrome to chronic obstructive pulmonary disease, asthma, and lung cancer [15]. Undoubtedly, the best possible solution to this problem is the development of new energy resources as alternatives to fossil fuels. However, even when fossil fuels are expected to be gradually replaced by new energy resources, their use is estimated to account for more than 50% by 2050 [16]. Therefore, there has been increasing interest in the development of functional substances that have protective effects on air-pollution-derived respiratory diseases [17].

Although it has been demonstrated that oral probiotics can affect the lung immune response based on the gut–lung axis, the evidence regarding their preventive effects against air-polluted particulate-matter-induced immune imbalance and neutrophilic inflammation is limited. Therefore, the purpose of this study was to screen beneficial lactic acid bacteria (LAB) based on in vitro anti-inflammatory efficacy and evaluate the respiratory protective effect of candidate probiotics in terms of modulating the inflammatory cell population using an animal model of ambient particulate matter (PM)-induced lung inflammation. The study observed that the candidate probiotics developed in this study exhibited local anti-inflammatory effects on intestinal inflammation by preventing a reduction of T_reg_ cells, as well as anti-inflammatory effects against PM-induced lung inflammation, including inhibition of neutrophil infiltration and pro-inflammatory cytokine expression. The results of this study could have important implications for the development of functional substances with protective effects on respiratory diseases derived from ambient PM.

## 2. Materials and Methods

### 2.1. Cell Culture and Preparation of Lactobacillus spp.

A human colon cell line (HT-29; No. 0038, KCBL, Seoul, Korea) was used to screen the functional *Lactobacillus* spp. strains with anti-inflammatory potency. The cells were maintained in Dulbecco’s Modified Eagle Medium (DMEM) supplemented with 2% (*v/v*) penicillin–streptomycin, 4-(2-hydroxyethyl)-1-piperazineethanesulfonic acid (HEPES), and 10% (*w/v*) fetal bovine serum (FBS). The medium and supplements were purchased from Gibco BRL (Gaithersburg, MD, USA). The cells were seeded at a density of 1 × 10^5^ in six-well culture plates and grown as adherent fmonolayers at 37 °C and 5% CO_2_ until a density of 1 × 10^6^ cells was achieved.

*L. rhamnosus* GG (LGG; ATCC53103, American Type Culture Collection, Manassas, VA, USA) and *Lactobacillus* isolates were cultured under anaerobic conditions (Whitley DG250, Don Whitley Scientific Ltd., Shipley, West Yorkshire, UK) at 37 °C for 48 h on de Man, Rogosa and Sharpe (MRS) agar (DF0881-17-5 and DF214010, BD, San Jose, CA, USA); subsequently, the representative colonies were picked from the plates and inoculated into fresh MRS broth at 180 rpm and 37 °C for 18–22 h (SI-600R, JEIOTech, Seoul, Republic of Korea). After centrifugation and washing with PBS, the cells were diluted to 1 × 10^8^ CFU/mL with DMEM.

### 2.2. mRNA Expression of Pro-Inflammatory Cytokine

The HT-29 cell monolayers were treated with *Lactobacillus* spp. strains at 37 °C for 6 h. The co-cultures of the HT-29 cells and each strain were washed twice with PBS and treated with lipopolysaccharide (LPS) (*Escherichia coli* O111:B4, Sigma-Aldrich, Saint Louis, MO, USA) for 16 h. The cells were then detached by treating with trypsin-EDTA (Gibco BRL, Gaithersburg, MD, USA). To evaluate the anti-inflammatory effects, the inflammatory cytokines TNF-α and IL-1β were selected as biomarkers. mRNA was extracted using a commercially available kit (Z6010; Promega, Madison, WI, USA), and its quantity and purity were determined using a nanophotometer (NP80; Implen GmbH, Munich, Germany); the ratio of absorbance at 260 nm and 280 nm ranged between 2.0 and 2.4 for all samples. Before synthesizing cDNA, treatment with DNase at 1 unit/µg RNA was performed to inhibit DNA contamination in the samples. cDNA was synthesized using equal concentrations of RNA with Murine Leukemia Virus (MLV) reverse transcriptase (Transcription kit; Enzynomics, Daejeon, Republic of Korea) and oligo (dT) (Qiagen Inc., Valencia, CA, USA). Real-time PCR was performed using SYBR Green PCR master mix (Bio-Rad, Hercules, CA, USA) in 10 μL reaction mixture with 0.5 μM primers on a CFX Connect™ Real-Time System detector. The PCR conditions were 50 °C for 2 min and 95 °C for 15 s, followed by 40 cycles at 95 °C for 15 s and 60 °C for 1 min. To calculate the relative quantification of gene expression compared with that of β-actin (the housekeeping gene), the results were expressed as ∆∆CT values. The raw CT values from the reactions were analyzed by a modified delta-Ct method with efficiency correction using a PCR data analysis program, q base, to obtain the relative quantification values.

### 2.3. Animal Care and Maintenance

The experiments were performed according to the protocols approved by the Institute Animal Care and Use Committee of the KT&G Central Research Center (KT&G19-002). After quarantine, the animals were acclimated for seven days prior to group isolation. During the entire study period, the mice were housed in a room under controlled conditions of temperature (20.5–23.2 °C), humidity (36.2–56.3%), and a 12 h dark/light cycle. Animals were isolated in stainless steel mesh cages (two to three mice per cage) with wood embedding and allowed access to rodent chow and filtered water ad libitum.

### 2.4. Anti-Inflammatory Effect of KC3 on Ear Edema Mouse Model

To evaluate the anti-inflammatory activity of KC3, a croton-oil-induced ear edema mouse model was used. The edema lesion area and gross lesion pattern were calculated to compare anti-inflammatory potency among different groups. Twenty-four 6-week-old male ICR mice (ORIENT Bio, Suwon, Korea) were purchased and divided into four groups (*n* = 6/group): (1) In the normal group, 0.5% carboxymethylcellulose sodium (CMC, 419273, Sigma-Aldrich, Saint Louis, MO, USA) was used as a vehicle for the control animals; (2) for the IND group, 10 mg/kg indomethacin (a cyclooxygenase inhibitor) was used as a positive control; and (3 and 4) the *L. plantarum* KC3 groups were treated with 200 and 400 mg/kg (1 × 10^9^ CFU and 2 × 10^9^ CFU, respectively). It was orally administered in a volume of 300 μL. One hour after administration on the fifth day, acetone or 2.5% croton oil (C0421; Tokyo Chemical Industry Co., Ltd., Tokyo, Japan) was applied to the right ear skin to induce ear edema. Under CO_2_ anesthesia, the ear thickness was measured using a thickness gauge (543-122-15; Digimatic, Mitutoyo Co., Ltd., Tokyo, Japan) and analyzed using the velocity change method. The edema inhibition rate was calculated by comparing the results of the negative control group (CRO, croton oil treatment alone) with those of the KC3 treatment groups.

### 2.5. Immune Regulatory Effect of KC3 in DSS-Induced Colitis Model

#### 2.5.1. Experimental Design

Twenty-four 6-week-old male C57BL/6 mice (ORIENT Bio, Suwon, Korea) were divided into four groups (*n* = 6/group). DSS (3% *w/v*, 36,000–50,000 kDa, MFCD00081551; MP Biomedicals, Inc., Irvine, CA, USA) was administered through drinking water ad libitum. The four groups were as follows: (1) non-treated control (normal), (2) DSS-treated alone (CTL, negative control), (3) DSS induction followed by 150 mg/kg 5-aminosalicylic acid treatment (5-ASA, positive control), and (4) DSS induction followed by 200 mg/kg KC3 (1 × 10^9^ CFU/head) (KC3). DSS was administered through drinking water for eight days in all groups except the normal group, in which the animals were provided only drinking water. The mouse necropsy was performed under isoflurane anesthesia.

#### 2.5.2. Measurement of Clinical Parameters

All animals were weighed daily, and the weight was expressed as the relative weight change compared with that on day 0. The clinical signs of colitis (weight loss, stool consistency, and fecal blood) were monitored daily for 30 min to calculate the disease activity index score and survival rate [18].

#### 2.5.3. Mesenteric Immune Cell Phenotyping Using Flow Cytometry

At necropsy, mesenteric lymph nodes (MLNs) were harvested, and the cells were isolated immediately in a sterilized dish on ice by homogenizing the tissue and passing the supernatant through a 70 μm strainer (352350, BD Falcon). The MLNs (10^7^ cells/mL) were incubated (30 min, 4 °C, darkness) with the indicated monoclonal antibodies in a BD FACSCanto™ II Flow Cytometry System. The proportions of CD4^+^CD69^+^ T cells, CD8^+^CD69^+^ T cells, CD19^+^CD69^+^ B cells, and CD4^+^Foxp3^+^ T_reg_ cells were calculated using fluorescence-activated cell sorting (FACS). All materials and antibodies used for flow cytometry were purchased from BD Biosciences (San Jose, CA, USA). Particularly, the CD4^+^Foxp3^+^ T_reg_ cells were stained intracellularly using the BD Cytofix/Cytoperm™ Fixation/Permeabilization Kit (Cat. No. 554714).

#### 2.5.4. Immunofluorescence Staining

The colon tissue section was floated on a superfrost slide (Fisherbrand™, Fisher Scientific, PA, USA) and washed thrice with PBS; the cells were then fixed in 4% paraformaldehyde for 10 min. After removing the fixative buffer solution, the tissues were incubated with fluorescent-labeled mouse anti-CD4+ (sc-13573 AF488; green) and anti-FOXP3+ (sc-53876 AF594; red) antibodies at 37 °C for 2 h. After rinsing with PBS, the cell nuclei were stained with Hoechst solution (sc-200908; blue). All antibodies were purchased from Santa Cruz Biotechnology (Dallas, TX, USA) and used at 1:500 dilution. Images were obtained using a laser scanning confocal microscope (LSM 800; Carl Zeiss, Jena, Germany). Image analysis software was used for automatic analysis, and the fluorochromes were visualized with the following excitation (Ex) and emission (Em) wavelengths: Hoechst, Ex 350 nm with Em 461 nm; AF488, Ex 490 nm with Em 525 nm; and AF594, Ex 590 nm with Em 617 nm.

#### 2.5.5. Histological Analysis

Large-intestinal tissues were fixed in 10% neutral buffered formalin for 24 h and then embedded in paraffin. For hematoxylin and eosin (H&E) staining, the paraffin-embedded tissue sections (4 μm) were dewaxed and rehydrated using xylene and ethanol and stained with H&E. The stained slides were analyzed using the VM600 Digital Slide Scanning System (Motic, CA, USA).

### 2.6. Respiratory Protective Effects of KC3 in Ambient PM-Induced Mouse Model

#### 2.6.1. Preparation of Ambient PM

The ambient PM (provided by KT&G Central Research Center, Daejeon, Republic of Korea) used in the study was dissolved in DMSO to obtain concentrations of 10 mg/mL coal-fired powder, 10 mg/mL fly ash, and 5 mg/mL DEP and then diluted with 1% aluminum hydroxide gel adjuvant (in 99% saline) to final concentrations of 1.5 mg/mL coal/fly ash and 0.75 mg/mL DEP [19].

#### 2.6.2. Experimental Design

Twenty-five 8-week-old male, BALB/c mice (ORIENT Bio, Suwon, Korea) were divided into five groups (*n* = 5/group) as follows: (1) 0.5% CMC oral administration and 1% aluminum hydroxide gel adjuvant intranasally injected group (NOR, normal control), (2) 0.5% CMC oral administration and ambient PM challenged in 1% alum (APM, negative control), (3) 3 mg/kg dexamethasone oral administration and ambient PM challenged in 1% alum (DEX, positive control 1), (4) 200 mg/kg *L. rhamnosus* oral administration and ambient PM challenged in 1% alum (LGG, positive control 2), and (5) 200 mg/kg *L. plantarum* KC3 oral administration and ambient PM challenged in 1% alum (KC3). Airway inflammation was induced by intranasal injection of the ambient PM with 1% aluminum hydroxide gel adjuvant to all groups except the NOR group, which was administered the vehicle only on days 4, 7, and 10. All animals were sacrificed under isoflurane anesthesia after 48 h of the last intranasal injection, and necropsy was performed under isoflurane anesthesia to collect samples. Blood was withdrawn by cardiac puncture and collected in EDTA Vacutainers (BD, San Jose, CA, USA) to analyze the complete blood count (CBC) using a Coulter counter (Beckman Coulter, Brea, CA, USA), and part of the blood was isolated from the peripheral blood mononuclear cells (PBMCs) using the BD FACSCanto™ II Flow Cytometry System.

#### 2.6.3. Chemokine and Pro-Inflammatory Cytokine Assays in the Bronchoalveolar Fluid

Bronchoalveolar fluid (BALF) was collected and analyzed following previously described methods [20]. Briefly, BALF was obtained by intranasal injection and aspiration with 1 mL PBS repeatedly for three times. Cells in the BALF were isolated by centrifugation at 3000× *g* for 3 min to identify the cell composition, and the supernatant was collected at −80 °C for cytokine analysis as described in previous studies. The concentrations of chemokines and cytokines associated with pro-inflammatory responses in the BALF, including chemokine (C-X-C motif) ligand (CXCL)-1, macrophage inflammatory protein (MIP)-2, IL-17, and TNF-α, were analyzed using an ELISA kit (R&D Systems, Minneapolis, MN, USA) according to the manufacturer’s instructions.

#### 2.6.4. Measurement of Neutrophil Infiltration in the BALF

To confirm the effect on the number of neutrophil cells on the total number of cells in the BALF, Diff-Quik Staining (1.11957; Sigma-Aldrich, Saint Louis, MO, USA) was performed according to the method described in the literature [21].

#### 2.6.5. Analysis of Composition of PBMCs and Cells Derived from the BALF and Lungs

To analyze the subpopulations of immune cells, including CD170 (Siglec F)^−^Gr-1^+^ neutrophils, CD11b^+^Gr-1^+^ cells, and CD4^+^ and CD8^+^ T cells, flow cytometry was used. The lungs were sterilized, harvested, and homogenized in a cell strainer. All antibodies used for flow cytometry were purchased from BD Biosciences (San Jose, CA, USA) except for the PerCP-eFlourTM 710-CD170 (Siglec F) antibody (eBioscience^TM^, invitrogen, 46-1702-82). Blood was collected from the heart using a 1 mL syringe containing 25 μL of heparin as an anticoagulant. The collected blood (200 μL) sample was mixed with 4.5 mL of Ammonium-Chloride-Potassium (ACK) lysing buffer solution (BP10-548E, Lonza Co., Basel, Switzerland) and incubated at room temperature for 2 min to lyse the RBCs, followed by centrifugation at 640× *g* for 5 min at 4 °C. The sediment was re-suspended in 4.5 mL of PBS and centrifuged as described above. The pellet was re-suspended in 1 mL of FACS buffer and divided into 250 μL samples. Then, 400 μL of FACS buffer was added, and the cells were washed and incubated with 2 μL of antibody for 30 min. After discarding the supernatant, 300 μL of the fixative solution was added and stored at 4 °C until further analysis.

### 2.7. Statistical Analysis

Continuous data including cytokine expression, immune cell population, weight gain, and colon length were analyzed with a one-way analysis of variance (ANOVA) followed by Tukey’s post hoc test. Discrete data such as the disease score were analyzed with Kruskal–Wallis H followed by post hoc testing using un-paired Mann–Whitney U tests.

## 3. Results

### 3.1. Screening of Potential Probiotic Lactobacillus Strains with Anti-Inflammatory Effects

**Figure 1:**
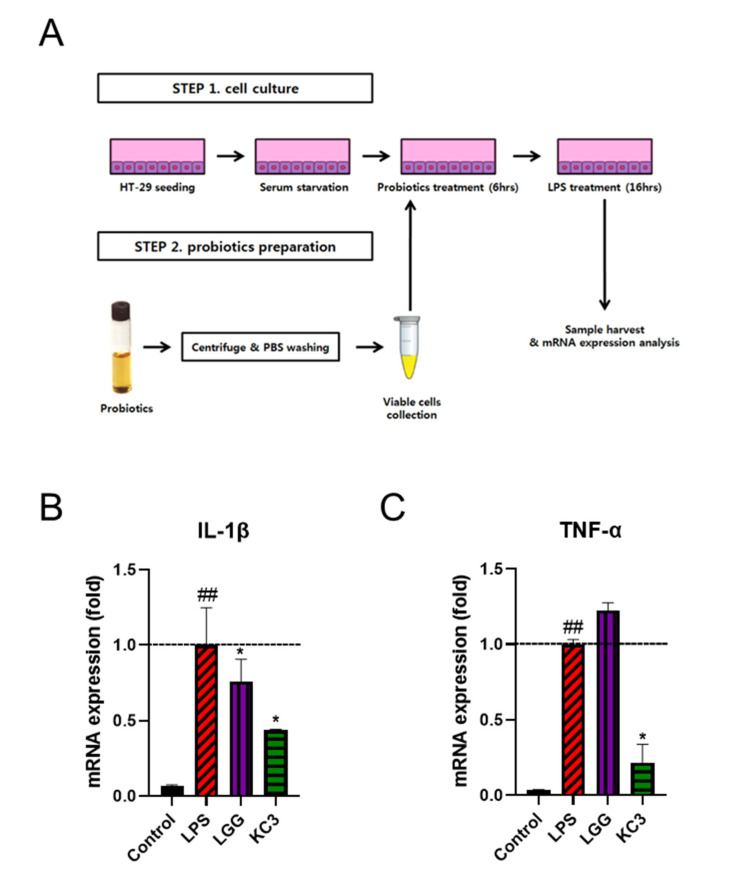
Anti-inflammatory effect of KC on LPS-induced inflammation. (**A**) Schematic of in vitro screening of lactic acid bacteria mRNA expression of pro-inflammatory cytokines of IL-1β (**B**) and TNF-α (**C**) in LPS-treated HT-29 cells. * Significant difference compared with the LPS control group (*p* < 0.05). ^##^ Significant difference compared with the PBS control group (*p* < 0.01).

To identify potential probiotic *Lactobacillus* strains with anti-inflammatory effects, a screening was conducted using LAB strains as shown in Figure 1A. Among the 19 strains tested, KC3 exhibited a repeated anti-inflammatory effect against lipopolysaccharide (LPS)-induced inflammation (Figure S1). Specifically, KC3 significantly decreased the expression of IL-1β and TNF-α compared with the LPS-treated control (*p* < 0.05, Figure 1B). Notably, KC3 exhibited an inhibitory effect of approximately 60% and 80% on the expression of IL-1β and TNF-α, respectively (Figure 1B,C). In comparison, the positive control *L. rhamnosus* GG (LGG) showed a significant inhibitory effect on the expression of IL-1β by 25%, while the expression of TNF-α by LGG was not significantly different from the LPS-treated control (Figure 1B,C).

**Figure 2:**
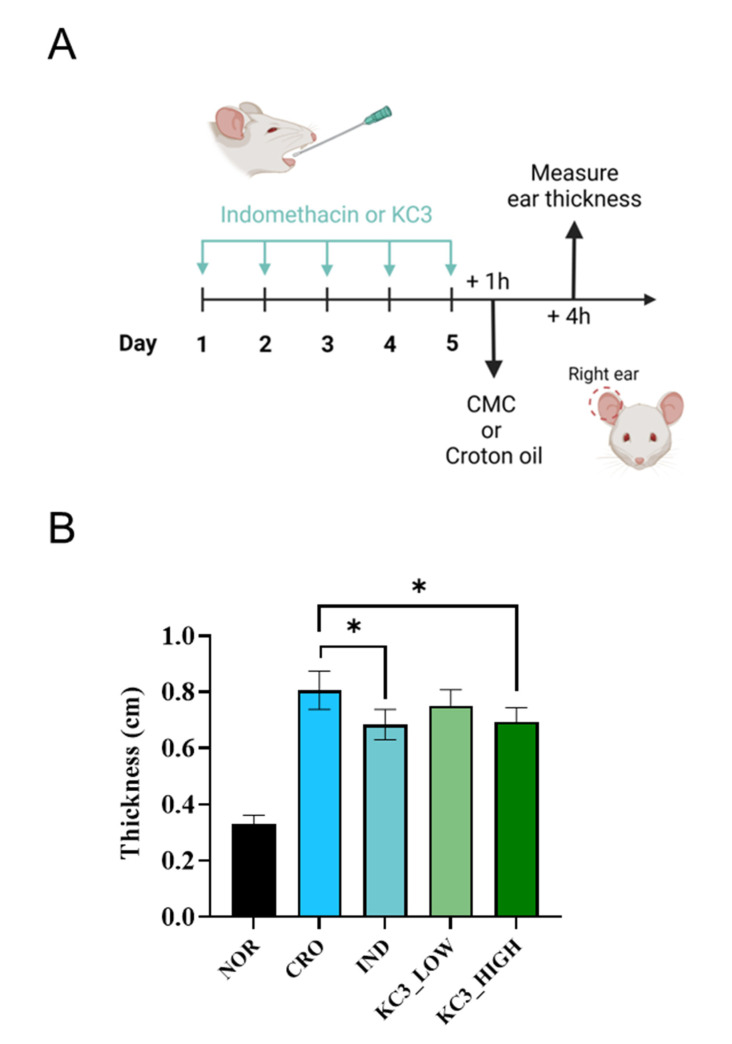
Ameliorating effect of KC3 on croton-oil-induced ear inflammation. (**A**) Schematic of anti-inflammatory evaluation using ear edema mouse model. Prophylactic treatment for five days was provided by oral gavage before applying croton oil. (**B**) Ear thickness was measured as an indicator of the severity of inflammation following croton oil application. IND, indomethacin. Data are represented as mean ± SD (*n* = 5 per group). * Significant difference compared with the croton-oil-treated control group (*p* < 0.05).

Based on the in vitro anti-inflammatory effect of KC3, further validation was conducted in vivo using a mouse ear edema model (Figure 2A). Croton oil was used to induce ear edema, resulting in an increase in ear thickness by approximately 250% compared with non-treated normal ear tissue (Figure 2B). KC3 at 400 mg/kg significantly inhibited the thickness of the ear by 23% compared with the control, which was a similar inhibitory effect to that of indomethacin at 10 mg/kg contributing to 25% inhibition (Figure 2B). Although KC3 at 200 mg/kg also inhibited the thickness of the ear by 11% compared with the control, statistical significance was not shown.

### 3.2. Anti-Inflammatory Effects of KC3 on DSS-Induced Colitis

#### 3.2.1. Clinical Observation and Macroscopic Intestinal Lesion

**Figure 3:**
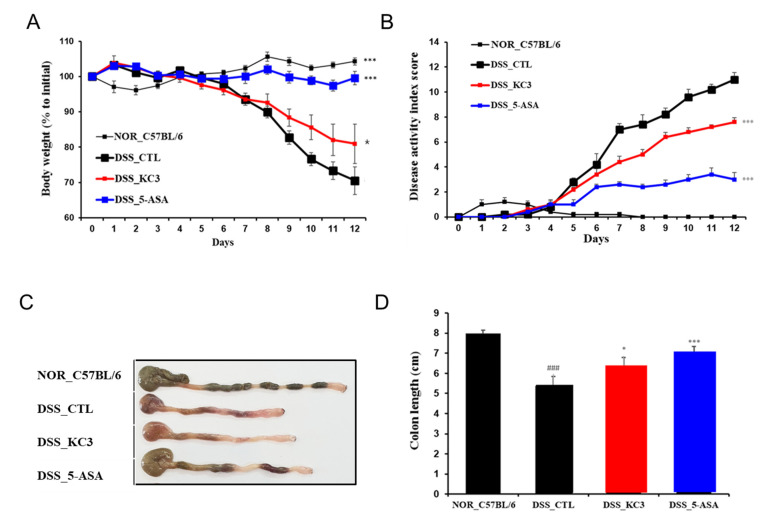
Protective effect of KC3 on DSS-induced colitis. The clinical assessment of the DSS-induced colitis model in mice was conducted through daily measurements of body weight (**A**) and disease activity score (**B**). A representative image of the colon is shown in (**C**), and the total length from the anus to the cecum was measured on the fourth day after DSS cessation (**D**). 5-ASA, 5-aminosalicylic acid. *, *** Significant difference compared with the DSS control group (*p* < 0.05, *p* < 0.01). ^###^ Significant difference compared with the normal group (*p* < 0.01). Data are represented as mean ± SD (*n* = 6 per group).

Since KC3 showed an inhibitory effect on pro-inflammatory cytokines and anti-inflammatory effects against ear edema, the anti-inflammatory effects of KC3 potentially colonizing the large intestine were investigated using a DSS-induced colitis model. DSS intake without other treatment resulted in significant weight loss from day 7 to the end of the experiment compared with non-DSS intake (Figure 3A). KC3 significantly improved body weight loss from day 9 to 12 after DSS water intake (Figure 3A). Moreover, KC3 also significantly decreased the disease activity index score compared with DSS intake without treatment (Figure 3B). In macroscopic evaluation, KC3 significantly inhibited the shortening of the length of the colon compared with DSS intake without treatment (Figure 3C,D). These results indicate that KC3 has the potential to mitigate DSS-induced colitis.

#### 3.2.2. Alteration of Intestinal Immune Cell Subtype by KC3 Treatment

**Figure 4:**
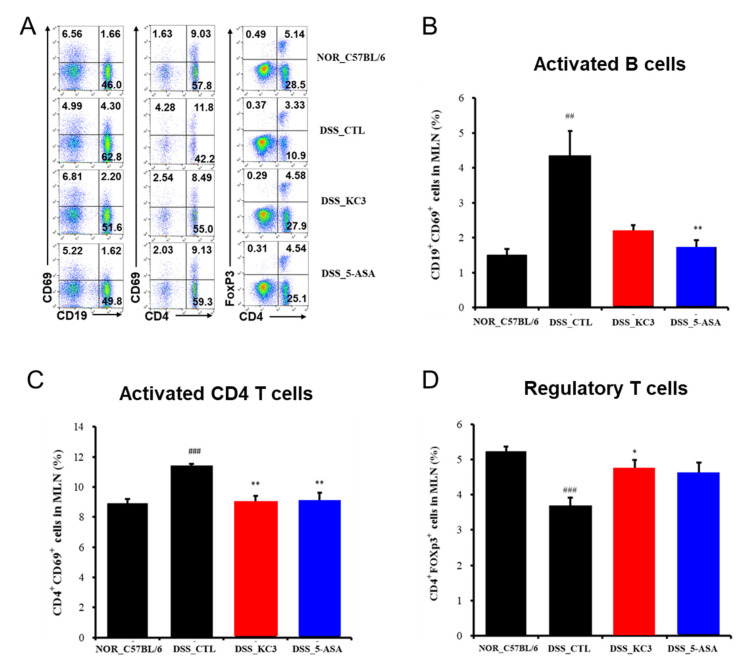
Immune cell phenotyping of mesenteric lymph nodes (MLNs) in mice of the DSS-induced colitis model. (**A**) Representative scatterplots of MLN flow cytometric immunophenotyping. Proportions of activated B cells (**B**), activated CD4^+^ T cells (**C**), and regulatory T cells (**D**) were measured using flow cytometry. The number on each box indicates the percentage of distinct population sorted by fluorescence signals. 5-ASA, 5-aminosalicylic acid. *, ** Significant difference compared with the DSS control group (negative control group, *p* < 0.05, *p* < 0.01). ^##^, ^###^ Significant difference compared with the normal group (*p* < 0.01, *p* < 0.001). Data are represented as mean ± SD (*n* = 5 per group).

To determine whether the anti-inflammatory effects of KC3 on DSS colitis were associated with the population of intestinal mucosal immune cells, an immunophenotypic analysis of the colon and mesenteric lymph node was performed (Figure 4A). The proportion of activated CD19^+^CD69^+^ B cells isolated from the mesenteric lymph nodes was also significantly higher in the DSS control group than that in the normal group (Figure 4B). However, no significant decrease in the ratio of activated B cells was observed following the treatment with KC3 (Figure 4B). The proportion of activated CD4^+^CD69^+^ T cells isolated from the mesenteric lymph nodes was significantly higher in the DSS control group than that in the normal group (Figure 4C). Treatment with KC3 significantly decreased the proportion of activated CD4^+^CD69^+^ T cells to the normal level (Figure 4C). In contrast to the activated T and B cells, the proportion of activated CD4^+^Foxp3^+^ T_reg_ cells was significantly lower in the DSS control group than that in the normal group (Figure 4D). Notably, treatment with KC3 significantly restored the proportion of activated CD4^+^Foxp3^+^ T_reg_ cells to the normal level (Figure 4D). These results suggest that KC3 can suppress the overactivation of CD4 T cells while simultaneously stimulating T_reg_ cells.

#### 3.2.3. Distribution of Regulatory T Cells in the Colon

**Figure 5:**
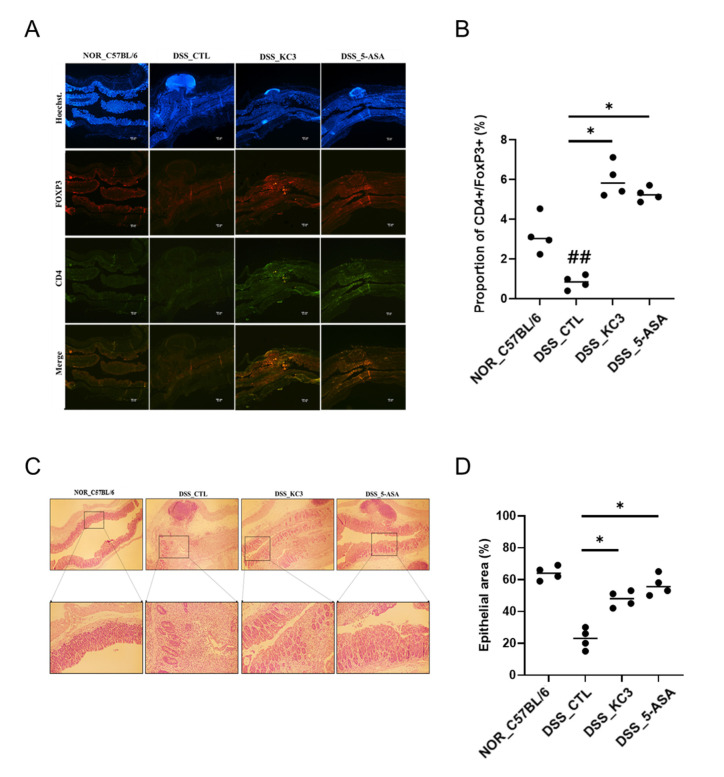
Distribution of T_reg_ cells and inflammatory lesions in colon tissue of mice of DSS-induced colitis. (**A**) Colonic T_reg_ cells were observed at 40× magnification by confocal microscopy after staining. To stain the nuclei for identifying the background tissue structure using Hoechst (blue), anti-CD4^+^ (Alexa Fluor 488, green), and anti-FOXP3^+^ (Alexa Fluor 594, red) merged images is regulatory T cell (CD4^+^FOXP3^+^) with yellowish. Scale bar is 100 μm. (**B**) Proportion of CD4^+^FOXP3^+^ positive area was calculated. (**C**) Histopathological lesion in colon tissue. The normal group showed the intact epithelial structure, whereas the DSS control group showed irregular epithelial lining with inflammatory cells and cell debris. Upper row shows 40× magnification; lower row shows 100× magnification. (**D**) The percentage of the area of epithelial cells in the colon tissue was quantified. KC3 treatment improved the epithelial condition by preventing inflammatory cell infiltration. 5-ASA, 5-aminosalicylic acid. * Significant difference compared with the DSS control group (*p* < 0.05). ^##^ Significant difference compared with the normal group (*p* < 0.01). Data are represented as mean ± SD (*n* = 5 per group).

To confirm an alteration of the T_reg_ cell distribution in the colon tissue by KC3 treatment, immunofluorescence staining was performed. The DSS control group showed a decrease in the fluorescence intensity of T_reg_ cells in the colon tissue compared with that in the normal group (Figure 5A,B). The KC3 treatment group showed an increase in the fluorescence intensity of T_reg_ cells compared with that in the DSS control group (Figure 5A,B). In the histopathological evaluation, the colon epithelial changes were evaluated based on the calculation of the intact epithelial area. The normal group contained more than 60% of the intact epithelial tissues, whereas the DSS control group contained only less than 25% of the intact epithelial area (Figure 5C,D). The KC3 treatment significantly restored the epithelial layer by 50% in colitis lesions (Figure 5C,D). These results suggest that treatment with KC3 can alleviate intestinal inflammation by promoting T_reg_ cells.

### 3.3. Anti-Inflammatory Effects of KC3 on Ambient PM-Induced Respiratory Disorder

#### 3.3.1. Expression Levels of Inflammatory Cytokines and Chemokines in the Bronchoalveolar Fluid (BALF)

**Figure 6:**
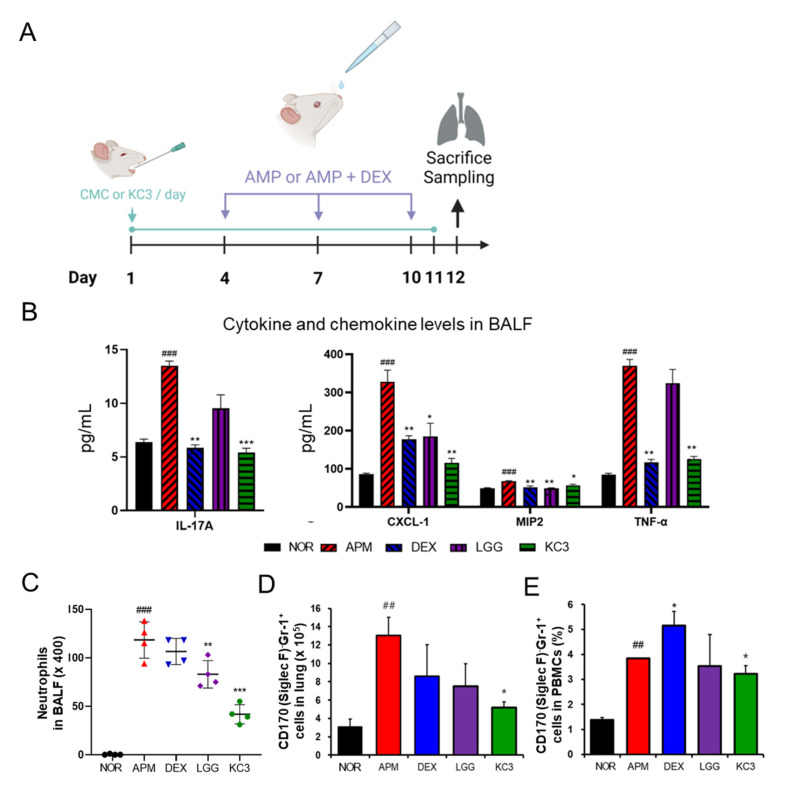
Anti-inflammatory effect of KC3 on ambient particulate matter (PM)-induced lung inflammation model. (**A**) Schematic of the experimental design. The PM was administered intranasally on days 4, 7, and 10. Treatment was conducted daily for 11 days by oral gavage. All mice were sacrificed on day 12 to harvest the BALF, lung tissues, and blood. The concentration of pro-inflammatory cytokine production (**B**) and the absolute number of neutrophils (**C**) were measured in the BALF. (**D**) The number of CD170 (Siglec F)^-^Gr-1^+^neutrophils were measured by flow cytometry in the lung tissues. (**E**) The proportion of lymphocyte and neutrophil in PBMC was measured by flow cytometry. APM and DEX represent ambient PM and dexamethasone, respectively. *, **, *** Significant difference compared with the ambient PM control group (*p* < 0.05, *p* < 0.01, *p* < 0.001). ^##^, ^###^ Significant difference compared with the normal group (*p* < 0.01, *p* < 0.001). Data are represented as mean ± SD (*n* = 5 per group).

Since recent research has shown that the mesenteric lymph nodes may play a role in regulating immune responses in other parts of the body including the lung, we hypothesized that promotion of T_reg_ cells in the mesenteric lymph nodes by KC3 treatment could contribute to the inhibition of lung inflammatory disorder. The ambient PM-induced lung inflammation model was established as shown in Figure 6A. In the BALF analysis, KC3 significantly reduced the levels of IL-17 (39.97%, *p* < 0.001), CXCL-1 (35.3%, *p* < 0.01), MIP-2 (84.43%, *p* < 0.05), and TNF-α (34.14%, *p* < 0.01), which were elevated by ambient PM exposure (Figure 6B).

#### 3.3.2. Identification of Immune Cell Subtypes in BALF and Lung Tissue

To evaluate the severity of lung inflammation, the differential count and proportion of the immune cell subtype was measured. The absolute number of neutrophils in the BALF was significantly reduced in the KC3 treatment group compared with that in the ambient PM control group. Moreover, KC3 significantly reduced the neutrophil number by 64.69% (*p* < 0.001) compared with that of the dexamethasone-treated control (Figure 6C).

The flow cytometry analysis showed that the KC3 treatment significantly reduced the number of CD8^+^ T cells in the BLAF compared with the ambient PM control (33.5%, *p* < 0.05, Figure S2A). Additionally, CD11b^+^Gr-1^+^, also known as myeloid-derived suppressor cells, was significantly decreased by the KC3 treatment compared with the ambient PM control (*p* < 0.05, Figure S2B). Similar to the pattern shown in the BALF, the KC3 treatment significantly reduced the number of CD170 (Siglec F)^-^Gr-1^+^ neutrophil and CD11b^+^Gr-1^+^ cell infiltration in the lung tissue compared with the ambient PM control (*p* < 0.05, Figure 6D and Figure S3A). However, no significant reduction of lymphocyte subsets by the KC3 treatment was shown in the lung tissue (Figure S3B). These results indicate that KC3 can inhibit the immune cell infiltration in the airway as well as the interstitial tissue.

#### 3.3.3. Identification of Immune Cell Subtypes in PBMC

The ambient PM control group showed an increase in the neutrophil of CD170 (Siglec F)^−^Gr-1^+^ in PBMCs (Figure 6E). The KC3 treatment significantly reduced the neutrophil proportion and elevated the lymphocyte proportion by 57.48% and 44.81%, respectively, compared with those in the ambient PM control group (Figure S4A, *p* < 0.01). In the analysis of lymphocyte subtypes, the ambient PM induced a reduction in the CD4^+^ T lymphocyte proportion without altering the CD8^+^ T lymphocyte proportion, and KC3 significantly increased the proportion of CD4^+^ T cells (10.71%, *p* < 0.05) compared with that in the ambient PM control group (Figure S4B). Similar to BALF and lung tissue, CD11b^+^Gr-1^+^ cell proportions were significantly reduced in the KC3 group compared with those in the ambient PM control group (Figure S4C).

## 4. Discussion

Probiotics can reduce local intestinal inflammation by directly reducing the production of pro-inflammatory cytokines in intestinal cells [22,23,24]. Recent studies have shown that probiotics can also have a systemic anti-inflammatory effect beyond their local effects by secreting anti-inflammatory molecules such as short-chain fatty acids, bacteriocins, and exopolysaccharides [25,26,27,28]. Given their potential for both local and systemic anti-inflammation, we developed probiotics with efficacy in both intestinal and lung inflammation. One candidate probiotic, KC3, showed the direct anti-inflammatory effect on intestinal cells by inhibiting pro-inflammatory cytokines and modulating T_reg_ cells in mesenteric lymph nodes. Additionally, KC3 successfully inhibited ambient PM-induced lung inflammation by preventing inflammatory cell infiltration and cytokine production in BALF and lung tissue.

Maintaining a homeostatic balance of the immune cell population is one of the key factors responsible for the anti-inflammatory effect of the gut microbiota [29]. Among all immune cells, T_reg_ cells are particularly closely related to gut inflammation [30]. The role of T_reg_ cells in gut inflammation has been demonstrated in previous studies using T_reg_ cell-depleted transgenic mice, which exhibit severe lesions such as intestinal inflammation [31,32]. In addition to genetically engineered mice, chemicals such as DSS can induce intestinal inflammation and cause the loss of T_reg_ cells, concurrent with severe gut disorders [33]. The present study showed that the protective effect of KC3 against DSS-induced colitis is accompanied by the restoration of T_reg_ cell populations while reducing CD4^+^ T cells in the mucosal lamina propria and MLNs. Similar results have been observed in previous studies investigating the effect of probiotics on gut inflammation. For instance, a previous study showed that *L. plantarum* CBT LP3 attenuated DSS-induced colitis by inducing T_reg_ cells in the peritoneum cavity, which contributed to inhibiting proinflammatory cytokine production [34]. Furthermore, the probiotic formulation including *Bifidobacterium* and *Lactobacillus* reduced the severity of experimental colitis by inducing T_reg_ cell population in MLNs, while concurrently increasing IL-2, IL-4, and IL-10 expression [35]. This evidence suggests that T_reg_ cell modulation by probiotics could play a significant role in reducing gut inflammation.

Beyond local intestinal anti-inflammatory effect, KC3 has also demonstrated systemic anti-inflammatory effects in vivo. Initially, we used the croton oil ear edema model to evaluate the anti-inflammatory properties of KC3 against acute inflammation. This model has been commonly used for screening compounds with anti-inflammatory efficacy [36,37]. Croton oil contains 12-O-tetradecanoylphorbol-13 acetate (TPA), which induces edema through the process of inflammatory cell infiltration into tissues [38]. IL-1β is a crucial cytokine that induces the TPA-derived inflammatory process by stimulating granulocyte development, neutrophil infiltration, and production of IL-17, a strong pro-inflammatory cytokine [38]. We could infer that KC3 had systemic inhibition against IL-1β production based on its anti-edema properties. As anticipated, we observed the inhibitory effects of KC3 on IL-1β and IL-17 in the BALF of the ambient PM-induced lung inflammatory model, which led to a reduction in the infiltration of inflammatory and immune-related cells in both the BALF and lung tissues.

Recent studies have shown that alterations in local immune responses derived from the gut microbiota can affect systemic immune status. The gut microbiota can interact with immune cells, including dendritic cells, to modulate the gut-associated lymphoid tissue, which helps maintain a balance between Th1/Th2 immunity and reduces systemic inflammation [39,40]. Moreover, the microbiota-derived metabolites can stimulate T_reg_ cells to regulate both the systemic immune status and the gut immune system [13]. In the present study, KC3 treatment ameliorated the ambient PM-induced lung inflammation, which might be due to its effect on the T_reg_ cell proliferation, leading to the suppression of inflammatory signals, including CXCL-1, IL-17, and TNF-α in the BALF. Notably, the inhibition of IL-17, a potent chemokine inducer in the BALF, contributed to the reduction of neutrophil infiltration, the main cause of lung inflammation and a prognostic indicator. Therefore, treatment with KC3 effectively maintained the homeostasis of the lung immune environment, from the parenchymal tissue to the alveolar space, as evidenced by similar alterations in both the lung homogenate and BALF cell population, resulting in a reduced proportion of neutrophils compared with that in the non-treated control.

Our findings suggest that KC3 could be a promising candidate for the development of functional foods or supplements aimed at reducing inflammation in both the intestinal and respiratory systems. The anti-inflammatory effects of KC3 were observed in various animal models and in vitro, indicating its potential for local and systemic anti-inflammatory activity. In particular, KC3 was able to modulate the immune cell population and reduce the levels of pro-inflammatory cytokines and chemokines, suggesting that it could promote immune homeostasis and prevent inflammation in both the gut and lung. Further studies are needed to fully elucidate the mechanisms underlying the anti-inflammatory effects of KC3 and to explore its potential for use in humans.

## Data Availability

Not applicable.

## Supplementary Materials

The following supporting information can be downloaded at: https://www.mdpi.com/article/10.3390/microorganisms11040967/s1, Figure S1: Pro-inflammatory cytokine gene expression; Figure S2: Immune cell subtypes in the BALF; Figure S3: Immune cell subtypes in the lung; Figure S4: Immune cell subtypes in PMBCs.
