# Supplementary material for: Protective Effect of Novel Lactobacillus plantarum KC3 Isolated from Fermented Kimchi on Gut and Respiratory Disorders
Source: Microorganisms. 2023 Apr 7;11(4):967. doi: 10.3390/microorganisms11040967 (PMC10141104; doi:10.3390/microorganisms11040967)
Supplement: Supplementary file 1 [file microorganisms-11-00967-s001.zip › microorganisms-2315630-supplementary.pdf]

Supplementary Figures

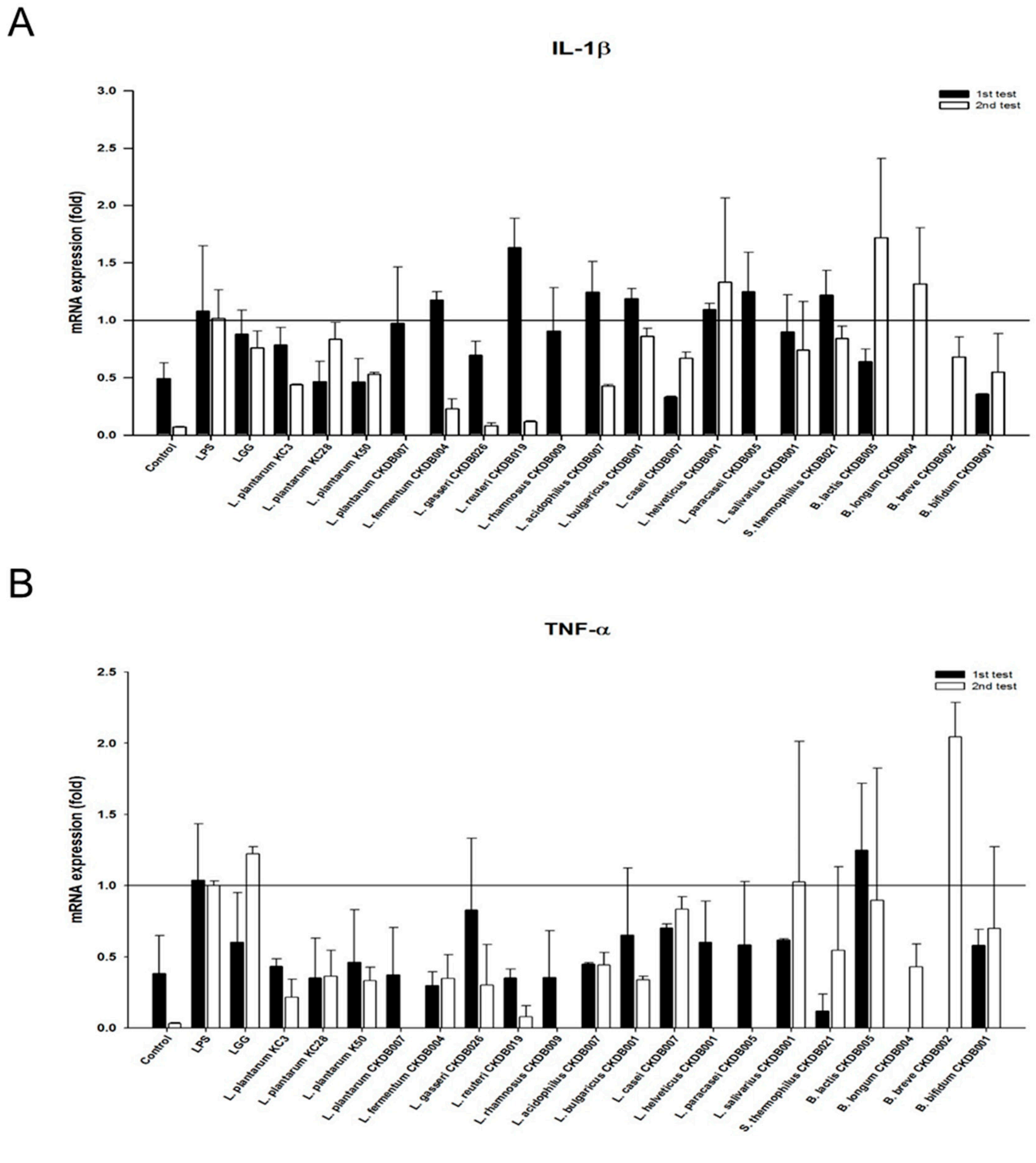

Figure S1. Screening of probiotics isolated from Kimchi based on inhibitory effect against IL-1β (A) and TNF-α (B) mRNA expression in LPS-induced inflammation.

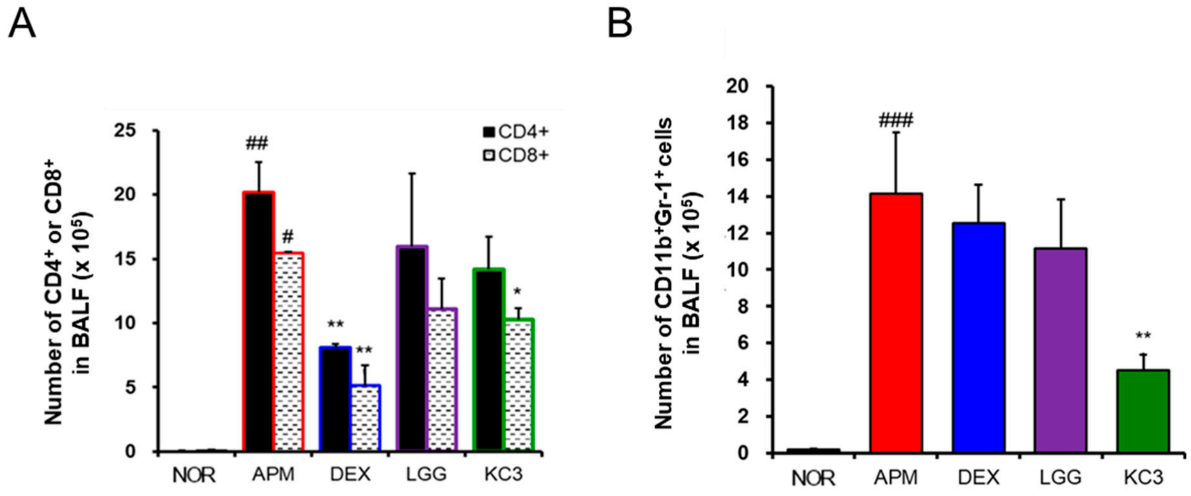

Figure S2. Population analysis of immune cell subtypes in the BALF. The absolute number of $CD4^+$ or $CD8^+$ (A), and $CD11b^+Gr\text{-}1^+$ (B) cells were measured by flow cytometry. $^{*, **}$Significant difference compared to the ambient PM control group ($p < 0.05$, $p < 0.01$). $^{\#, \#\#, \#\#\#}$Significant difference compared to the normal group ($p < 0.05$, $p < 0.01$, $p < 0.001$).

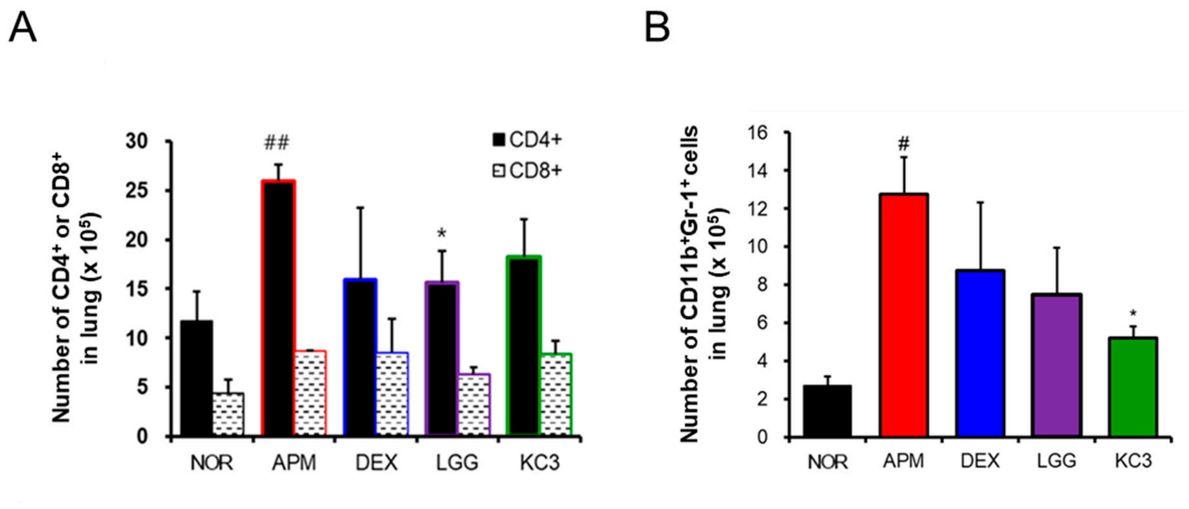

Figure S3. Population analysis of immune cell subtypes in the lung tissue. The absolute number of CD4^{+} or CD8^{+} (A), and CD11b^{+}Gr-1^{+} (B) cells were measured by flow cytometry. *Significant difference compared to the ambient PM control group ($p < 0.05$, $p < 0.01$). $^{\#,\ \#\#}$Significant difference compared to the normal group ($p < 0.05$, $p < 0.01$).

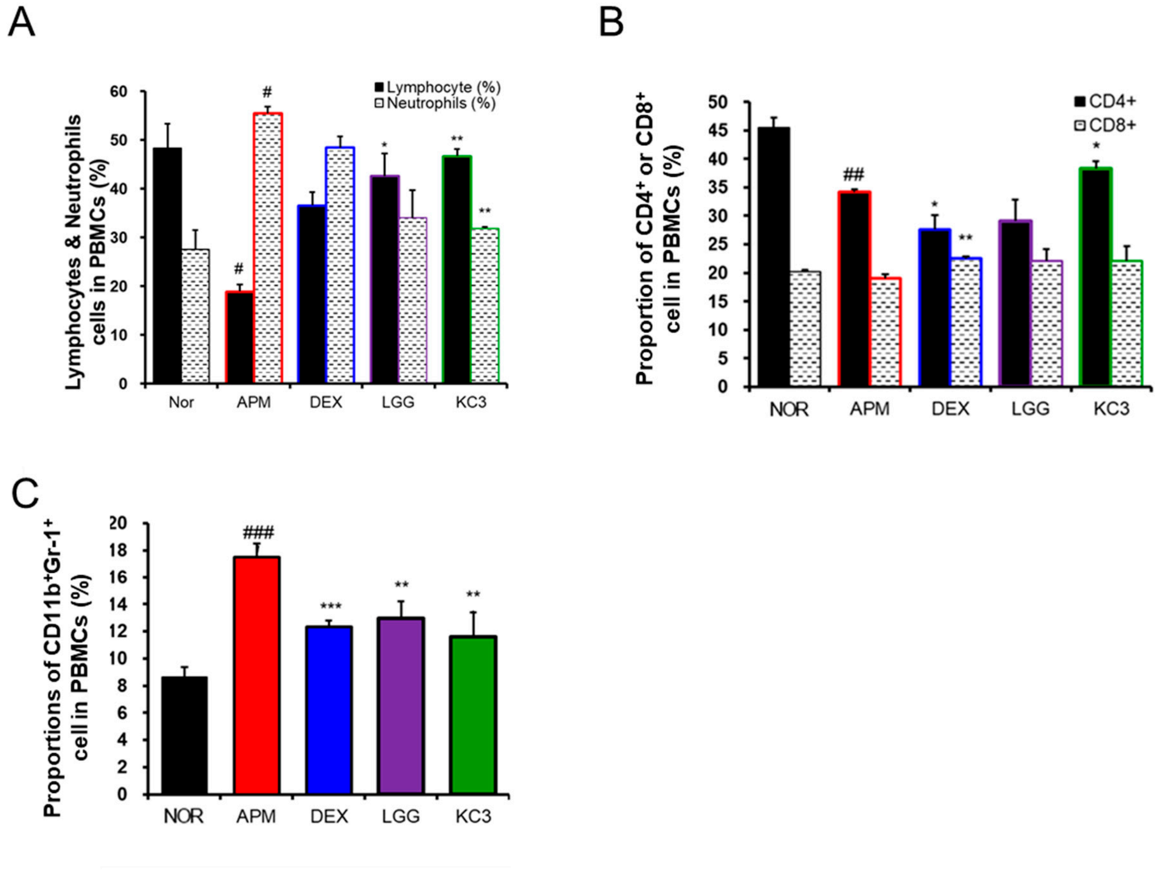

Figure S4. Population analysis of immune cell subtypes in PBMCs. The proportion of lymphocyte or neutrophils (A), CD4^{+} or CD8^{+} (B), and CD11b^{+}Gr-1^{+} (C) cells were measured by flow cytometry. *,**,***Significant difference compared to the ambient PM control group (p < 0.05, p < 0.01, p < 0.001). $^{\#\#,\ \#\#\#}$Significant difference compared to the normal group (p < 0.01, p < 0.001).
